# Pneumococcal pneumonia coinfection in critically ill patients with influenza a (h1n1) primary viral pneumonia

**DOI:** 10.1186/2197-425X-3-S1-A829

**Published:** 2015-10-01

**Authors:** FJ González De Molina, A Rodríguez, S Barbadillo, E Díaz, J Solé-Violán, L Socias, JC Vergara, R Zaragoza, B Suberviola, L Vidaur, R Ferrer, I Martín-Loeches, SEMICYUC/GETGAG Working Group

**Affiliations:** Mutua Terrassa University Hospital, Terrassa, Spain; Hospital Universitari de Tarragona Joan XXIII, Tarragona, Spain; Hospital General de Cataluña, Sant Cugat del Valles, Spain; ParcTaulí Hospital, Sabadell, Spain; Hospital Dr. Negrín, Las Palmas, de Gran Canaria Spain; Hospital Son Llatzer, Mallorca, Spain; Hospital de Cruces, Vizcaya, Spain; Hospital Dr. Peset, Valencia, Spain; Hospital Universitario de Santander, Santander, Spain; Hospital de Donostia, San Sebastián, Spain; Mútua Terrassa University Hospital, Intensive Care, Terrassa, Spain; St James's University Hospital, Dublin, Ireland

## Introduction

It has been generally believed that influenza virus infection increase susceptibility to invasive pneumococcal pneumonia. However, information regarding the impact of *S. pneumoniae* respiratory coinfection (SpCoI) in patients affected with A(H1N1) primary viral pneumonia is scarce.

## Objectives

Our aim was to analyse the demographic and clinical differences between patients admitted to ICU due to A(H1N1) pneumonia with and without SpCoI.

## Methods

Prospective, observational, multicenter study conducted in 148 Spanish ICUs. Individuals with A(H1N1) confirmed using RT-PCR during the 2009 to 2014 influenza seasons were included and compared with those patients with SpCoI. Patients´ demographic, clinical, radiologic features, laboratory values, ICU and hospital length of stay (LOS) and outcomes were recorded. Discrete variables are expressed as counts (percentage) and continuous variables as medians with 25th to 75th interquartile range (IQR). Differences between groups were assessed using the x2 test and the Fisher exact test for categoric variables and Mann-Whitney U test for continuous variables.

## Results

Of 2343 patients with confirmed A(H1N1) pneumonia, 208 were excluded due to respiratory coinfection by non-pneumococal microorganism. At all, 1949 patients with A(H1N1) pneumonia were compared to 186 patients with documented SpCoI (incidence= 8.7%). Patients SpCoI presented a higher APACHE II score (14 [10-19] vs 17 [12-21.5], P = 0.001) and SOFA score (5 [3-8] vs 6 [4-9], P = 0.002). Patient with SpCoI were less obese (36.8% vs 22.2%, P < 0.001). HIV presented SpCoI more frequently (2.0% vs 4.3%, P = 0.036). No other differences in comorbidities were observed. Patients who had coinfection developed acute kidney injury more frequently (22.8% vs 32.4%, P = 0.004) but no difference in renal replacement therapy was observed (10.8% vs 11.5%, P= 0.773). Shock requiring vasopressors were more frequently in those patients with SpCoI (49.7% vs 61.1%, P = 0.003). No differences were observed in invasive mechanical ventilation (68.8% vs 65.0%, P = 0.211), prone positioning (18% vs 15.1%, P = 0.331) or LOS. Coinfection was not associated with increased ICU mortality (19.5% v 21.0%, P = 0.627).

## Conclusions

Incidence of SpCoI among Influenza virus infection admitted to ICU was low. There was no difference in LOS or mortality for patients with SpCoI despite their greater severity and organ dysfunction at ICU admission.
